# Assessment of Nasopharyngeal Cancer in Young Patients Aged ≤ 30 Years

**DOI:** 10.3389/fonc.2019.01179

**Published:** 2019-11-06

**Authors:** Yi Zhu, Xinmao Song, Ruichen Li, Huatao Quan, Li Yan

**Affiliations:** Department of Radiation, Eye and ENT Hospital, Fudan University, Shanghai, China

**Keywords:** young nasopharyngeal cancer, TNM stage, metastasis, radiation, SEER

## Abstract

**Purpose:** This study assessed the prognosis of young patients with nasopharyngeal cancer (NPC), both domestic and foreign, using data from the Surveillance, Epidemiology and End Results (SEER) database, a population-based database, and departmental records.

**Methods:** Patients diagnosed with NPC from 2004 to 2016 in the SEER database were reviewed. Young patients aged ≤30 years when diagnosed, were compared with older patients. The medical records of patients with NPC aged ≤30 years in our own department were also reviewed. The prognostic effects of different variables, including age, sex, race, tumor stage, pathology, radiation, and chemotherapy were assessed by Kaplan–Meier and Cox analyses.

**Results:** A total of 212 foreign and 257 domestic patients, with mean ages of 21.5 and 22.9 years and median ages of 22 and 24 years, respectively, were included. In SEER database, younger NPC patients had a more advanced tumor stage (74.5% stage III/IV vs. 63.1% in older patients) and a worse pathological differentiation, but a better prognosis (*P* < 0.0001). Interestingly, the younger the patient, the better the prognosis. Younger patients received a higher proportion of chemotherapy. M stage, overall stage and radiation were the primary prognostic factors. Similar and more comprehensive results were found in our center. Patients with distant metastases, at a primary visit, had worse diagnoses. Intensity-modulated radiotherapy and three-dimensional conventional radiotherapy had minimal effects on survival in young patients in our center. Most patients from our department received chemotherapy, and different induction chemotherapy regimens resulted in similar survival prognoses.

**Conclusions:** Young patients with NPC usually present at an advanced stage, but have a better overall prognosis. How to treat patients with metastasis is critical for improving prognoses in young NPC patients.

## Introduction

Nasopharyngeal cancer (NPC), which has a unique global distribution, is an epithelial tumor that originates from the pharyngeal recess of the nasopharynx (1). More than 80% of patients with NPC are from Southern China and Southeast Asia (2). NPC has diverse age distributions in different areas; for most low-incidence countries, NPC increases gradually with age. However, in high-incidence areas, NPC incidences reach peak levels between the ages of 40–59 years (3). The etiology of NPC is unique and multifactorial, involving genetic, epigenetic and environmental factors, such as Epstein–Barr virus infection (4). NPC in young patients has not yet been extensively studied, and definitive guidelines are still lacking. Similarly, the clinical characteristics and prognoses in these young patients with NPC have not been well-studied.

The Surveillance, Epidemiology, and End Results (SEER) program is a huge population-based database. It provides detailed information on disease incidence, prevalence, and survival (5). This study evaluated and compared the survival data of patients with NPC aged no more than 30 years, with the data of those aged over 30 years, in the SEER database. The study also compared treatment and prognostic parameters between patients in the database with those in hospital (EENT Hospital, Fudan University, Shanghai, China) and explored comprehensive parameters related to the prognosis of young patients with NPC.

## Methods

### Data Acquisition

SEER (Incidence-SEER 18 Regs Custom Data) data were obtained. Patients with NPC were carefully reviewed, and those with the following criteria were excluded: (1) diagnosis not confirmed by a histological examination and (2) not the first primary tumor. The patients were then classified into young (≤30 years) and older groups (>30 years) using age at the time of diagnosis. The survival of the two groups and influencing factors were analyzed, including age, sex, race, state, insurance, marital status, pathology, TNM stage, primary site, and chemoradiotherapy. NPC- and non-NPC-specific survival statuses were obtained from the SEER database. Tumor TNM staging was restaged based on the AJCC Staging Manual (Eighth Edition).

The data from young patients with NPC, aged no more than 30 years, in the EENT Hospital, Fudan University from 2008 to 2017, were retrospectively analyzed and compared with those from the SEER database. Parameters influencing survival, including age, sex, pathology grade, TNM stage, radiation, chemotherapy, and Epstein–Barr virus (EBV) infection status were analyzed. The results of serum immunoglobulin A (IgA) antibodies to early antigen, capsular antigen, nuclear antigen 1, and Zta protein levels before treatment were reviewed, and patients with one or more IgA's exceeding reference values were regarded as EBV positive. Only 232 patients were included in the survival analyses, since 25 patients without any follow-up were excluded. The Ethics Committee of the EENT Hospital approved this study.

### Statistical Analysis

All analyses were performed using R software (v3.4.5; R Foundation for Statistical Computing, Vienna, Austria) and SPSS 24.0 (IBM, Inc., NY, USA). The baseline distributions in the two groups were compared using *t*, Wilcoxon, and chi-square tests. Kaplan–Meier and log-rank test analyses were used to construct and compare the cumulative survival curve of each patient variable. Univariate and multivariate Cox analyses were used to identify significant prognostic factors for the entire cohort. A *P* value < 0.05 (two-tailed) indicated a statistically significant difference.

## Results

### Clinicopathological Presentation in the SEER Database

A total of 3,962 patients were identified in the SEER database, according to the criteria for survival analysis from 2004 to 2016. In total, 212 enrolled patients were aged ≤30 years, while 3,750 were aged over 30 years at diagnosis, with mean ages of 21.5 and 56.9 years, and a median age of 22 and 56 years, respectively. As shown (Table 1), statistical differences were found for age, race, pathological differentiation, tumor stage, T stage, N stage, chemotherapy and marital status between young and old groups (*P* < 0.05). Young patients with NPC had a more advanced stage (both T and N stages), worse pathological differentiation and a higher proportion of chemotherapy.

**Table 1:** Patient demographic and clinical data from SEER.

		**≤30 years of age**	**>30 years of age**	*P* value^*^
Total No.		212	3,750	
Age (mean±SD)		21.5 ± 5.6	56.9 ± 12.3	**<0.001**
Age (median)		22 (18–26)	56 (48–65)	
Race	White	105 (49.5%)	1,979 (52.8%)	**<0.001**
	Black	53 (25.0%)	430 (11.5%)	
	Others	54 (25.5%)	1,341 (35.8%)	
Sex	Male	145 (68.4%)	2,722 (72.6%)	0.184
	Female	67 (31.6%)	1,028 (27.4%)	
Site	Superior wall	2 (0.9%)	42 (1.1%)	0.448
	Posterior wall	28 (13.2%)	421 (11.2%)	
	Lateral wall	11 (5.2%)	325 (8.7%)	
	Anterior wall	1 (0.5%)	43 (1.1%)	
	Overlapping	10 (4.7%)	151 (4%)	
	NOS	160 (75.5%)	2,768 (73.8%)	
Grade	G1-2	9 (4.2%)	552 (14.7%)	**<0.001**
	G3	51 (24.1%)	1,405 (37.5%)	
	G4	92 (43.4%)	597 (15.9%)	
	Gx	60 (28.3%)	1,196 (31.9%)	
Stage	I	8 (3.8%)	245 (6.5%)	**0.013**
	II	26 (12.3%)	739 (19.7%)	
	III	64 (30.2%)	977 (26.1%)	
	IVA	76 (35.8%)	1,016 (27.1%)	
	IVB	18 (8.5%)	370 (9.9%)	
	Unknown	20 (9.4%)	403 (10.7%)	
Tstage	T1	42 (19.8%)	1,026 (27.4%)	**0.042**
	T2	42 (19.8%)	805 (21.5%)	
	T3	47 (22.2%)	714 (19.0%)	
	T4	61 (28.8%)	821 (21.9%)	
	Tx	20 (9.4%)	384 (10.2%)	
Nstage	N0	27 (12.7%)	851 (22.7%)	**<0.001**
	N1	53 (25.0%)	1,230 (32.8%)	
	N2	82 (38.7%)	1,015 (27.1%)	
	N3	41 (19.3%)	439 (11.7%)	
	Nx	9 (4.2%)	215 (5.7%)	
Mstage	M0	182 (85.8%)	3,154 (84.1%)	0.777
	M1	18 (8.5%)	370 (9.9%)	
	Mx	12 (5.7%)	226 (6%)	
Radiation	No	186 (87.7%)	3,340 (89.1%)	0.547
	Yes	26 (12.3%)	410 (10.9%)	
Chemotherapy	No	30 (14.2%)	649 (17.3%)	0.236
	Yes	182 (85.8%)	3,101 (82.7%)	
Insurance	No	22 (10.4%)	798 (21.3%)	**<0.001**
	Yes	190 (89.6%)	2,952 (78.7%)	
	Unknown	12 (5.7%)	147 (3.9%)	**0.041**
Marry	No	165 (77.8%)	1,332 (35.5%)	
	Yes	37 (17.5%)	2,200 (58.7%)	
	Unknown	10 (4.7%)	218 (5.8%)	

* *Bold values means statistical significance*.

### Clinicopathological Presentation at the EENT Hospital

A total of 257 young patients, aged ≤30 years were identified between 2008 and 2017. The included patients had a mean age of 22.9 years and a median age of 24 years. As Table 2 shows, among all the young patients with NPC, male patients accounted for 71.6% and female 28.4%. The main pathological type of nasopharyngeal carcinoma in China is non-keratinizing and undifferentiated squamous cell carcinoma; this type was represented in up to 91.1% of cases in the center. Up to 87.6% of enrolled young patients with NPC were in an advanced stage (stage III or IV). The EBV infection status was recorded in only 164 patients, most of whom were diagnosed after 2011, of whom 126 (76.8% in patients with recorded EBV status) were EBV positive.

**Table 2:** Patient demographic and clinical data from our center.

**Characteristics**		**All patients**		**Patients included in survival analyses**	
		**No**.	**%**	**No**.	**%**
**No**.		257	100	232	100
Age (mean ± SD)		22.9 ± 5.2		23.0 ± 5.2	
Age (median)		24 (19–27)		24 (19–27)	
Sex	Male	184	71.6	163	70.3
	Female	73	28.4	69	29.7
Grade	NUSC	234	91.1	210	90.5
	Other	23	8.9	22	9.5
T stage	T1	36	14.0	35	15.1
	T2	49	19.1	39	16.8
	T3	95	37.0	89	38.4
	T4	77	29.9	69	29.7
N stage	N0	19	7.4	18	7.8
	N1	70	27.2	62	26.7
	N2	146	56.8	132	56.9
	N3	22	8.6	20	8.6
M stage	M0	249	96.9	227	97.8
	M1	8	3.1	5	2.2
Stage	I	6	2.3	6	2.6
	II	26	10.1	23	9.9
	III	132	51.4	120	51.7
	IVA	85	33.1	78	33.6
	IVB	8	3.1	5	2.2
EBV status	No	38	14.8	38	16.4
	Yes	126	49.0	126	54.3
	Unknown	93	36.2	68	29.3
Chemotherapy	No	34	13.2	27	11.6
	Yes	221	86.0	203	87.5
	Unknown	2	0.8	2	0.9
IC	No	34	13.2	28	12.1
	Yes	221	86.0	202	86.0
	Unknown	2	0.8	2	0.9
CC	No	66	25.7	57	24.6
	yes	187	72.8	172	74.1
	Unknown	4	1.5	3	1.3
AC	No	83	32.3	75	32.3
	Yes	173	67.3	156	67.2
	Unknown	1	0.4	1	0.4
EAR	CR	188	73.2	172	74.1
	PR	53	20.6	47	20.2
	Unknown	16	6.2	13	5.6
Radiation	IMRT	197	76.7	191	82.3
	3D–CRT	54	20.0	36	15.5
	Both	6	2.3	5	2.2

*NUSC; non-keratinizing and undifferentiated squamous cell carcinoma; IC: induction chemotherapy; CC: concurrent chemotherapy; AC: adjuvant chemotherapy; EAR: response after radiation*.

All patients were treated with radiation; 76.7% received intensity-modulated radiotherapy (IMRT) while 20.0% received three-dimensional conventional radiotherapy (3-DCRT). Most patients were treated with chemotherapy; 86.0, 72.7, and 67.3% of patients received induction chemotherapy (IC), concurrent chemotherapy (CC), and adjuvant chemotherapy (AC), respectively. The induced chemotherapy regimens included PF (cisplatin + 5-Fu), GP (gemcitabine + cisplatin), TP (docetaxel + cisplatin), and TPF (docetaxel + cisplatin + 5-Fu). Single-drug cisplatin (25 mg/m^2^) was used concurrently with radiotherapy. Following radiation, 73.2% of patients achieved complete responses, without any residual tumor according to imaging after radiation.

### Survival Analysis of Patients From the SEER Database

**Figure 1:**
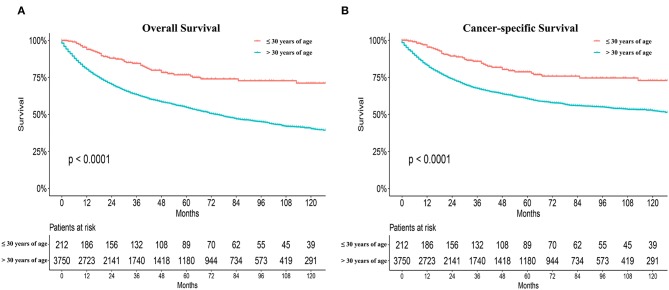
Overall survival (OS) and cause-specific survival between old and young patients from the SEER database **(A,B)**.

**Figure 2:**
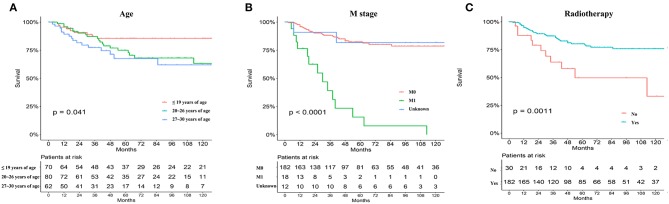
The prognosis of young NPC patients ≤ 30-year old stratified by age subdivision **(A)**, metastasis or not **(B)**, primary treatment mode (radiation vs. surgery) **(C)** from the SEER database.

Significant differences in overall survival (OS) and cause-specific survival were found between old and young patients (*P* < 0.0001, Figure 1). The 5-year survival was 76.8 ± 3.3% for young patients and 54.7 ±0.9% for the remaining cohort. Young patients were further divided into three groups: patients ≤19 years, 20–26 years, and 27–30 years. The younger the patient, the better the prognosis (*P* = 0.041, Figure 2A). Univariate analyses revealed that no distant metastasis and radiation therapy as initial treatment resulted in significantly better prognoses for young patients with NPC (Figures 2B,C). Kaplan–Meier estimates of OS by race, sex, marital status, insurance, primary site, histology grade, T/N stage and chemotherapy of young patients with NPC, showed no statistically significant differences (Figures S1, S2). Overall, radiotherapy is an effective treatment for young patients with NPC. Even at more advanced stages and with worse pathological differentiation, the survival of young patients with NPC was still better than that of older patients. The presence or absence of metastases, at first visit, significantly influenced prognoses.

### Survival Analysis of Patients at the EENT Hospital

**Figure 3:**
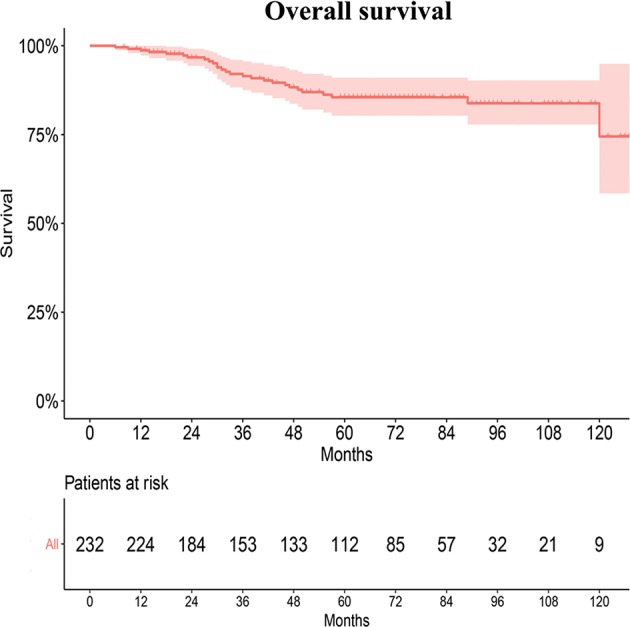
Survival analyses of young NPC patients in our radiation center.

Twenty-five patients without any follow up were excluded from the survival analyses. In our radiation department, the 5- and 10-year OS rates of young patients were 87.8 ± 2.4% and 86.0 ± 2.9%, respectively (Figure 3). However, the survival rates of patients in the SEER database and the departments could not be directly compared because of different baseline characteristics and tumor histopathology.

**Figure 4:**
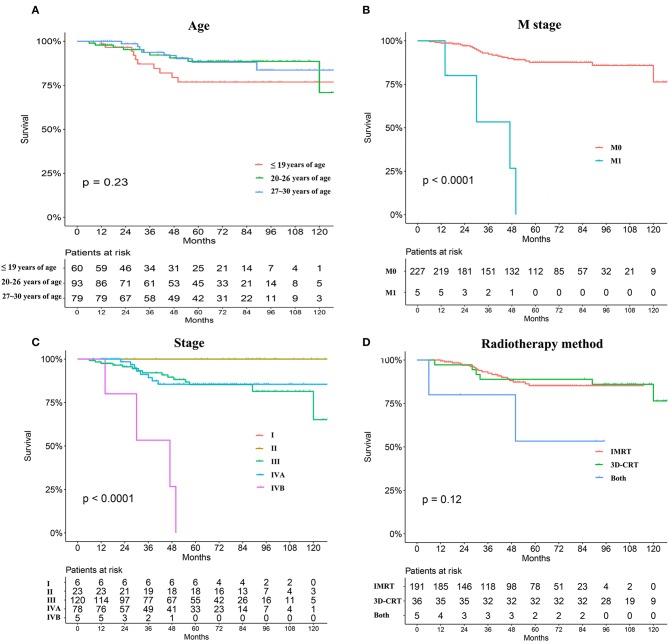
Survival analyses stratified by age subdivision **(A)**, metastasis or not at primary presentation **(B)**, TNM stage **(C)**, and radiation mode (IMRT vs. 3-DCRT) **(D)**.

Young patients were also divided into three groups according to age as described earlier, but differences in OS were not apparent (Figure 4A). Causes of death were grouped into two categories: recurrence (26.9%) and distant metastasis (76.9%). Recurrence occurred in 14 patients during the last clinical review; 12 patients experienced primary recurrence and two experienced cervical lymph node recurrence. Distant metastases, primarily in the liver, lung, and bone, were observed in 31 patients, including eight with metastasis at initial presentation. Recurrence and metastasis occurred mainly within 5 years, especially at 3 years after the end of treatment. Only two patients were found to have a relapse 5 years after treatment. Univariate analyses revealed that M and TNM stages significantly influenced the prognosis of young patients with NPC (*P* < 0.05, Figures 4B,C). No statistically significant difference was found in the OS of young patients with NPC between IMRT and 3-DCRT (*P* = 0.12, Figure 4D). Sex, pathology differentiation, T/N stage, EBV status, dose of radiotherapy and chemotherapy were not associated with prognosis (Figures S3, S4). The difference in IC regimens, including PF, TF, TPF and GP, did not change the long-term survival of patients (Figure S4). In conclusion, young patients had very good prognoses when compared with those from the SEER and AJCC databases. The TNM stage (only M stage) was significantly associated with prognosis, and the radiotherapy mode was not an independent prognostic factor for young patients with NPC. However, information regarding alternatives such as chemotherapy regimens and radiation doses is lacking.

## Discussion

There is no well-accepted standard when we refer to young cancer patients. In our manuscript, we chose 30 years of age as the threshold. Young patients with head and neck cancer account for <10% of all patients in SEER database. However, many studies have indicated a rise in the incidence of head and neck cancer in young individuals (6–8). It is generally believed that young patients with tumors tend to have worse prognoses due to tumor biology, including a more aggressive tumor at a later stage. Young patients with breast cancer tend to have worse survival when compared with older cohorts (9–12). Younger patients with laryngeal cancer have more advanced disease and poor OS when compared with older counterparts (13). Older patients with advanced oral or oropharyngeal cancers have similar survival rates as young patients; however, older patients have higher rates of therapy-related toxicities (14). The increase in the numbers of cancer patients with poor prognoses has increased the overall cancer burden in society. Therefore, it is of great value to realize age-specific differences in frequent cancers, and explore better treatments for younger adults.

An estimated 42,100 new NPC cases occurred in China in 2013, accounting for 1.14% of all new cancer cases (15). Age-specific incidence and mortality increases between the ages of 25 and 39 years, peaking at different ages and varying by location (15, 16). However, studies on younger cancer patients are limited, and definitive clinical guidelines are lacking. This study aimed to understand the incidence, survival and prognostic factors of young patients with NPC. Combining clinical information from home and abroad made the analysis clearer and persuasive.

An analysis of the SEER database indicated that when compared with the remaining cohort, young patients with NPC suffered more advanced disease; 74.5% of patients were classified as stage III/IV. The stage difference was apparent mainly in T and N stages, but not in M stage. Similar results were obtained in our department; 85.21% of patients were in stage III/IV and 3.1% of patients exhibited metastasis at initial presentation. It was unclear why both foreign and domestic young patients had more advanced stages. Young patients often overlook earlier symptoms, resulting in later detection. This could also be due to different characteristics and etiologies, at different ages. Despite the late clinical stage, the prognosis of young patients with NPC was better than that of older patients, according to the SEER database (5 year OS, 76.2 ± 3.5% vs. 54.2 ± 1.0%, respectively). Interestingly, in patients aged ≤ 30 years, from the SEER database, the younger the patient, the better the prognosis. It was presumed that younger patients had a better prognosis in China. However, in the department, data from patients aged over 30 were not available. The reason was that ~6,000 patients aged over 30 were identified in the same period, and no comparisons were made. The disease characteristics and the tumor biology, especially the EBV status of Asian patients with NPC, are quite different from those of Western patients. Therefore, whether younger patients had better prognoses in China still requires validation.

Recurrence and distant metastases are currently the main reasons for treatment failures in patients with NPC (17, 18). In our center, distant metastasis in the liver, lungs, and bone was observed in 31 patients; eight patients initially presented with metastasis. The probability of recurrence and metastasis in young patients with NPC, after 5 years of treatment was low. An associated study showed that a good prognosis of young patients with NPC could be attributed to more intensive treatments received by these patients, and their ability to bear more adverse toxic side effects. Also, young patients receive more family and financial support (19). The analysis of patients from the SEER database revealed that the proportion of patients who received radiotherapy, between the two groups of patients was similar. Young patients received a higher proportion of chemotherapy (89.0 vs. 78.8%, *P* < 0.001). In our center, 86.0% of young patients with NPC, also received a high intensity of chemotherapy, including 86.0% IC, 72.7% CC, and 67.3% AC. The survival advantage was not a result of chemotherapy intensity (Figures S2F, S4A). However, statistically significant differences in chemotherapy were not expected in these analyses, due to limitations in the small sample size and imbalanced groups. The intensity and cycle of chemotherapy also needs to be further explored in young patients with NPC.

Combining chemotherapy and radiotherapy is a mainstay strategy for improving the long-term outcome of patients with NPC (20, 21). Analysis of the SEER database showed that 12.5% of young patients did not receive radiation for the first treatment, while patients undergoing radiation showed improved rates of survival. With the increased use of IMRT, a higher local control rate and a better survival were achieved for one patient with NPC. In a large-sample study by Peng et al. (22), investigating 616 patients with NPC, the 5-year actuarial local control rate and the 5-year OS were 90.5 and 79.6%, respectively, in the IMRT group. These values were significantly higher than those in the conventional radiation therapy group (23). In a meta-analysis, including 13,304 patients with NPC, Du et al. (24), reported that the IMRT group was associated with a better 5-year OS (OR = 1.70; 95% CI = 1.36–2.12), tumor local control including local–regional free survival (OR = 2.08; 95% CI = 1.82–2.37), and progression-free survival (OR = 1.40; 95% CI = 1.26–1.56). IMRT is also associated with lower radiation toxicity, such as xerostomia, hearing loss, trismus and temporal lobe neuropathy. In our department, IMRT (76.65%) and 3-DCRT (23.35%) had minimal effects on the long-term survival of young patients with NPC (*P* = 0.897). One limitation of the present study was the lack of follow-up regarding long-term radiotherapy-related side effects of IMRT and 3-DCRT.

## Conclusions

This study comprehensively analyzed the prognosis of NPC in young patients, both at home and abroad. Young patients with NPC usually present at an advanced stage, but have a better overall prognosis. However, whether younger patients had better prognoses in other countries still need to be validated. How to prevent and treat patients with metastatic NPC is critical to improving the prognosis of these young patients with NPC.

## Data Availability Statement

The datasets generated for this study are available on request to the corresponding author.

## Ethics Statement

The studies involving human participants were reviewed and approved by Ethics Committee of EENT Hospital. Written informed consent was exempt by the ethic committee.

## Author Contributions

YZ, RL, and LY: conception and design. XS, HQ, and LY: administrative support. YZ and LY: provision of study materials or patients. YZ, XS, RL, and LY: collection and assembly of data. YZ, HQ, and LY: data analysis and interpretation. All authors: manuscript writing and final approval of manuscript.

### Conflict of Interest

The authors declare that the research was conducted in the absence of any commercial or financial relationships that could be construed as a potential conflict of interest.

## Supplementary Material

Figure S1 Kaplan-Meier estimates of OS by race **(A)**, sex **(B)**, marital status **(C)**, and insurance **(D)** for young patients with NPC from the SEER database.

Figure S2 Kaplan-Meier estimates of OS by primary site **(A)**, histology grade **(B)**, T stage **(C)**, N stage **(D)**, total stage **(E)**, surgery **(F)**, and chemotherapy **(G)** for young patients with NPC from the SEER database.

Figure S3 Kaplan-Meier estimates of OS by sex **(A)**, histology grade **(B)**, T stage **(C)**, and N stage **(D)** with NPC from our center.

Figure S4 Kaplan-Meier estimates of OS by chemotherapy **(A)**, induction chemotherapy **(B)**, adjuvant chemotherapy **(C)**, and EBV status **(D)** with NPC from our center.

## References

[1] Wang J Kang M Qin YT Wei ZX Xiao JJ Wang RS. Sp1 is over-expressed in nasopharyngeal cancer and is a poor prognostic indicator for patients receiving radiotherapy. Int J Clin Exp Pathol. (2015) 8:6936–43. 26261581 PMC4525915

[2] Cao SM Simons MJ Qian CN. The prevalence and prevention of nasopharyngeal carcinoma in China. Chin J Cancer. (2011) 30:114–9. 10.5732/cjc.010.10377 21272443 PMC4013340

[3] Qiu WZ Peng XS Xia HQ Huang PY Guo X Cao KJ. A retrospective study comparing the outcomes and toxicities of intensity-modulated radiotherapy versus two-dimensional conventional radiotherapy for the treatment of children and adolescent nasopharyngeal carcinoma. J Cancer Res Clin Oncol. (2017) 143:1563–72. 10.1007/s00432-017-2401-y 28342002 PMC5504129

[4] Xu T Huang Z Deng Y Wang S Su B Wei W. Clinical implications of hepatitis B viral infection in Epstein-Barr virus-associated nasopharyngeal carcinoma. J Clin Virol. (2015) 64:64–71. 10.1016/j.jcv.2014.11.024 25728081

[5] Zhan C Yang X Song X Yan L Radiotherapy vs surgery for T1-2N0M0 laryngeal squamous cell carcinoma: a population-based and propensity score matching study. Cancer Med. (2018) 7:2837–47. 10.1002/cam4.1525 PMC6051150 29733513

[6] Muscat JE Wynder EL. Tobacco, alcohol, asbestos, and occupational risk factors for laryngeal cancer. Cancer-Am Cancer Soc. (1992) 69:2244–51. 10.1002/1097-0142(19920501)69:9<2244::AID-CNCR2820690906>3.0.CO;2-O 1562970

[7] Macfarlane GJ Boyle P Scully C. Oral cancer in Scotland: changing incidence and mortality. BMJ. (1992) 305:1121–3. 10.1136/bmj.305.6862.1121 1463946 PMC1883702

[8] Depue RH. Rising mortality from cancer of the tongue in young white males. N Engl J Med. (1986) 315:647. 10.1056/NEJM198609043151013 3736606

[9] Owrang M Copeland RJ Ricks-Santi LJ Gaskins M Beyene D Dewitty RJ. Breast cancer prognosis for young patients. In Vivo. (2017) 31:661–8. 10.21873/invivo.11109 28652435 PMC5566918

[10] Kim JK Kwak BS Lee JS Hong SJ Kim HJ Son BH. Do very young Korean breast cancer patients have worse outcomes? Ann Surg Oncol. (2007) 14:3385–91. 10.1245/s10434-006-9345-9 17899295

[11] Maggard MA O'Connell JB Lane KE Liu JH Etzioni DA Ko CY. Do young breast cancer patients have worse outcomes? J Surg Res. (2003) 113:109–13. 10.1016/S0022-4804(03)00179-3 12943818

[12] Vollmer RT. Younger women with breast carcinoma have a poorer prognosis than older women. Cancer-Am Cancer Soc. (1996) 78:1518–9. 10.1002/(SICI)1097-0142(19961001)78:7&lt;1518::AID-CNCR24&gt;3.0.CO;2-1 8839562

[13] Nachalon Y Alkan U Shvero J Yaniv D Shkedy Y Limon D. Assessment of laryngeal cancer in patients younger than 40 years. Laryngoscope. (2018) 128:1602–5. 10.1002/lary.26951 29076536

[14] Biswas R Halder A Ghosh A Ghosh SK. A comparative study of treatment outcome in younger and older patients with locally advanced oral cavity and oropharyngeal cancers treated by chemoradiation. South Asian J Cancer. (2019) 8:47–51. 10.4103/sajc.sajc_7_18 30766854 PMC6348780

[15] Wei KR Zheng RS Zhang SW Liang ZH Ou ZX Chen WQ. Nasopharyngeal carcinoma incidence and mortality in China in 2010. Chin J Cancer. (2014) 33:381–7. 10.5732/cjc.014.10086 25096544 PMC4135367

[16] Li Y Chen QY Tang LQ Liu LT Guo SS Guo L Concurrent chemoradiotherapy with or without cetuximab for stage II to IVb nasopharyngeal carcinoma: a case-control study. BMC Cancer. (2017) 17:567 10.1186/s12885-017-3552-6 28836950 PMC5571586

[17] Sun J Huang Z Hu Z Sun R. Benefits of local tumor excision and pharyngectomy on the survival of nasopharyngeal carcinoma patients: a retrospective observational study based on SEER database. J Transl Med. (2017) 15:116. 10.1186/s12967-017-1204-x 28558725 PMC5450381

[18] Zhu Q Cao SM Lin HX Yang Q Liu SL Guo L. Overexpression of acylglycerol kinase is associated with poorer prognosis and lymph node metastasis in nasopharyngeal carcinoma. Tumour Biol. (2016) 37:3349–57. 10.1007/s13277-015-4148-x 26443540 PMC4844630

[19] Leu Y Chang Y Lee J Lo A Chen Y Chen H Prognosis of nasopharyngeal carcinoma in the elderly is worse than in younger individuals–experience of a medical institute. Int J Gerontol. (2014) 8:81–4. 10.1016/j.ijge.2013.08.008

[20] Sun XS Liu SL Luo MJ Li XY Chen QY Guo SS The development of radiotherapy, image technology and chemotherapy prolongs the survival of patients with nasopharyngeal carcinoma: a cohort study with 20305 patients from 1990 to 2012. Int J Radiat Oncol Biol Phys. (2019) 105:581–90. 10.1016/j.ijrobp.2019.06.2549 31319091

[21] Qiu WZ Huang PY Shi JL Xia HQ Zhao C Cao KJ. Neoadjuvant chemotherapy plus intensity-modulated radiotherapy versus concurrent chemoradiotherapy plus adjuvant chemotherapy for the treatment of locoregionally advanced nasopharyngeal carcinoma: a retrospective controlled study. Chin J Cancer. (2016) 35:2. 10.1186/s40880-015-0076-9 26739148 PMC4704429

[22] Peng G Wang T Yang KY Zhang S Zhang T Li Q. A prospective, randomized study comparing outcomes and toxicities of intensity-modulated radiotherapy vs. conventional two-dimensional radiotherapy for the treatment of nasopharyngeal carcinoma. Radiother Oncol. (2012) 104:286–93. 10.1016/j.radonc.2012.08.013 22995588

[23] Hui EP Ma BB Leung SF King AD Mo F Kam MK Randomized phase II trial of concurrent cisplatin-radiotherapy with or without neoadjuvant docetaxel and cisplatin in advanced nasopharyngeal carcinoma. J Clin Oncol. (2009) 27:242–9. 10.1200/JCO.2008.18.1545 19064973

[24] Du T Xiao J Qiu Z Wu K. The effectiveness of intensity-modulated radiation therapy versus 2D-RT for the treatment of nasopharyngeal carcinoma: A systematic review and meta-analysis. PLoS ONE. (2019) 14:e219611. 10.1371/journal.pone.0219611 31291379 PMC6619803

