# Supplementary figures and images for: Assessment of Nasopharyngeal Cancer in Young Patients Aged ≤ 30 Years
Source: Front Oncol. 2019 Nov 6;9:1179. doi: 10.3389/fonc.2019.01179 (PMC6851239; doi:10.3389/fonc.2019.01179)

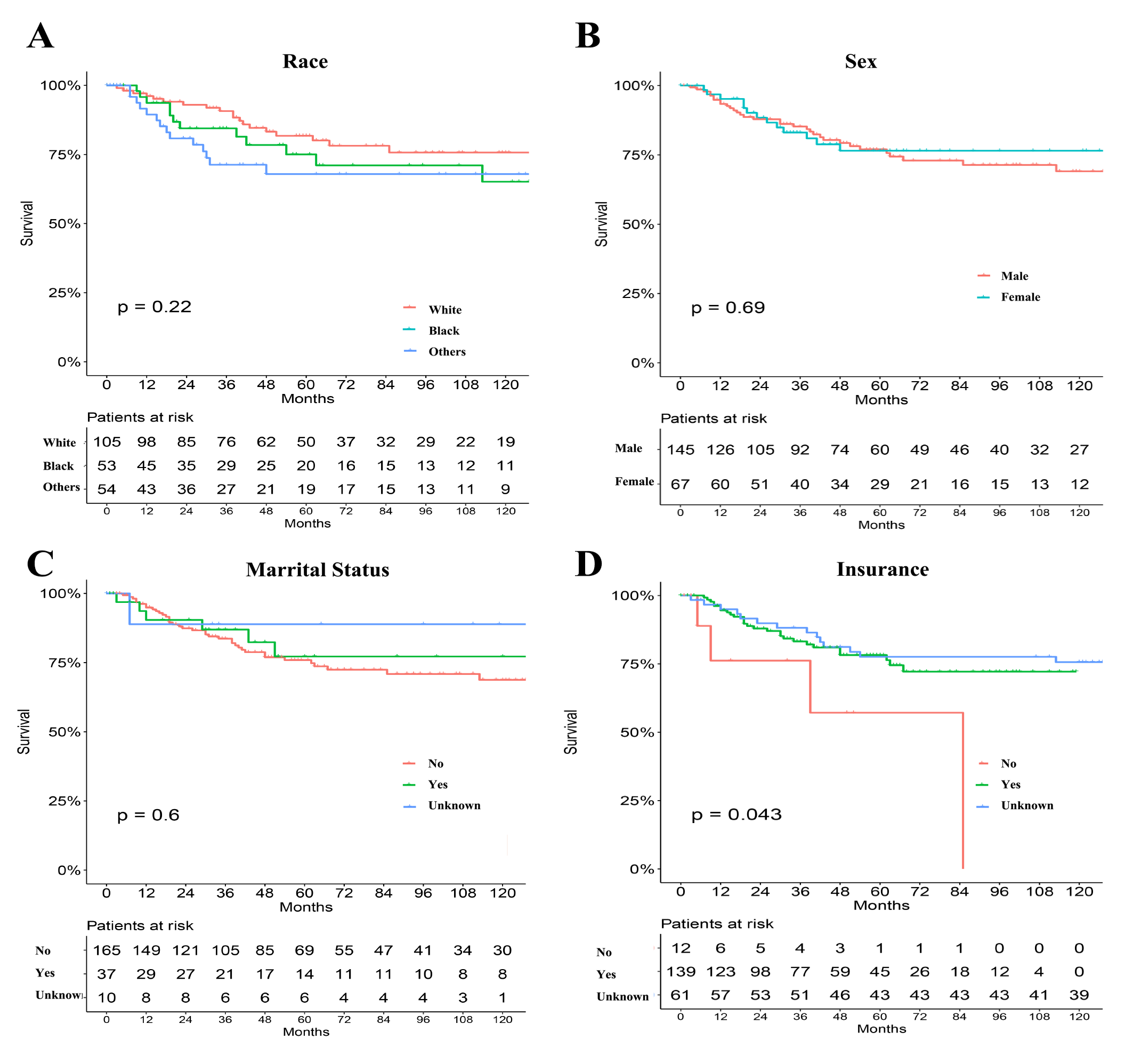

Supplement: Figure S1 — Kaplan-Meier estimates of OS by race (A), sex (B), marital status (C), and insurance (D) for young patients with NPC from the SEER database. [file Image_1.TIF]

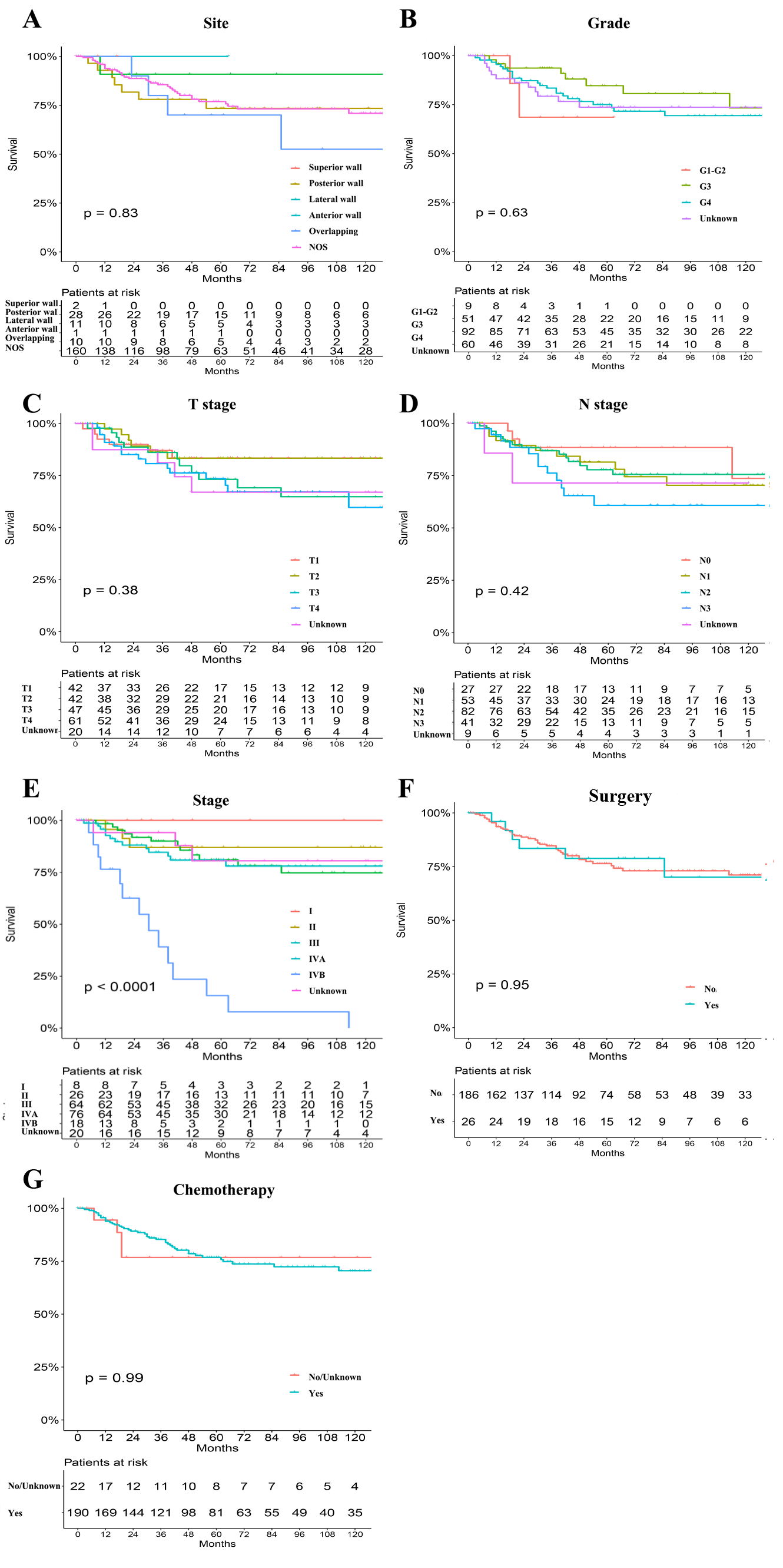

Supplement: Figure S2 — Kaplan-Meier estimates of OS by primary site (A), histology grade (B), T stage (C), N stage (D), total stage (E), surgery (F), and chemotherapy (G) for young patients with NPC from the SEER database. [file Image_2.TIF]

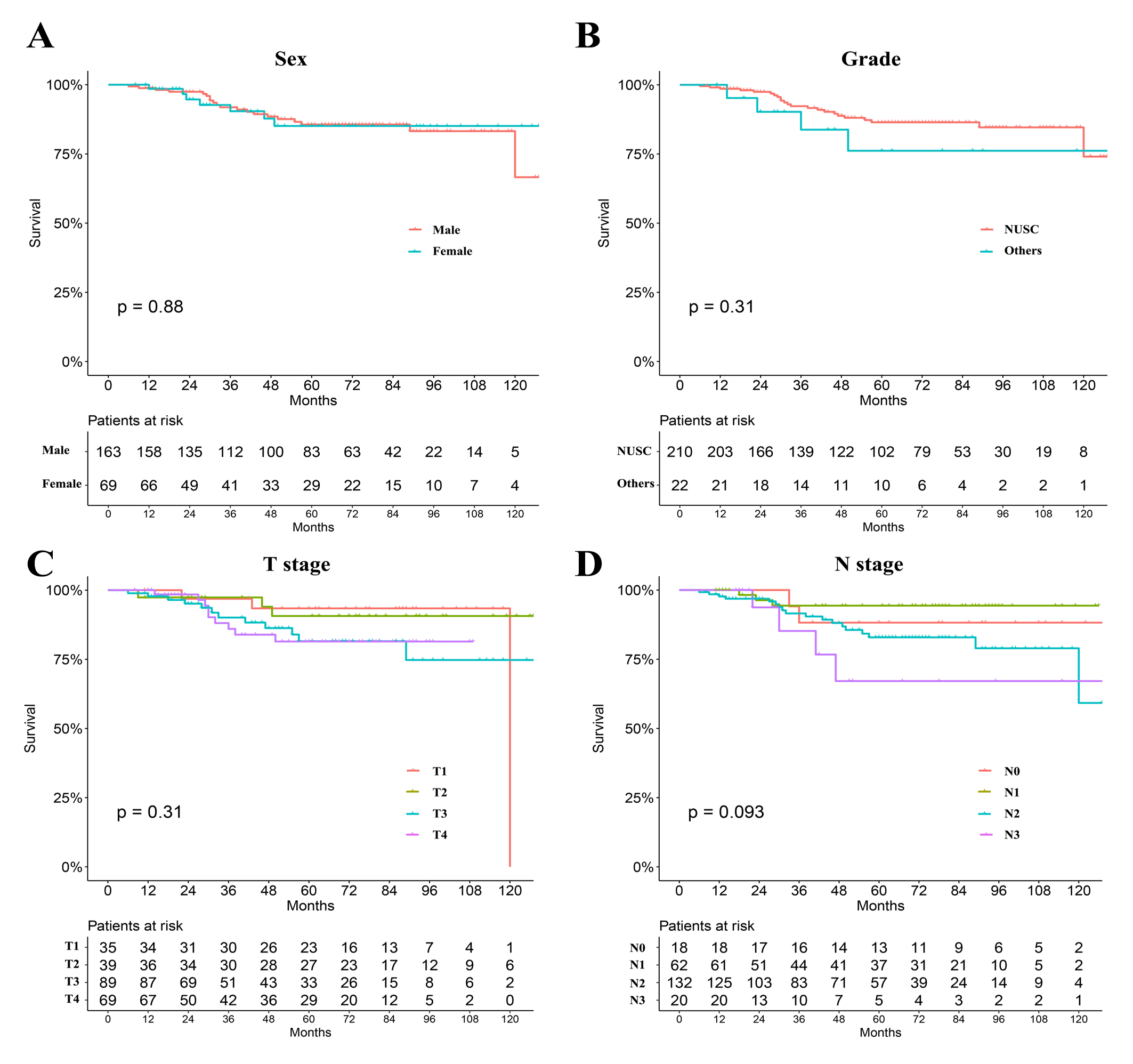

Supplement: Figure S3 — Kaplan-Meier estimates of OS by sex (A), histology grade (B), T stage (C), and N stage (D) with NPC from our center. [file Image_3.TIF]

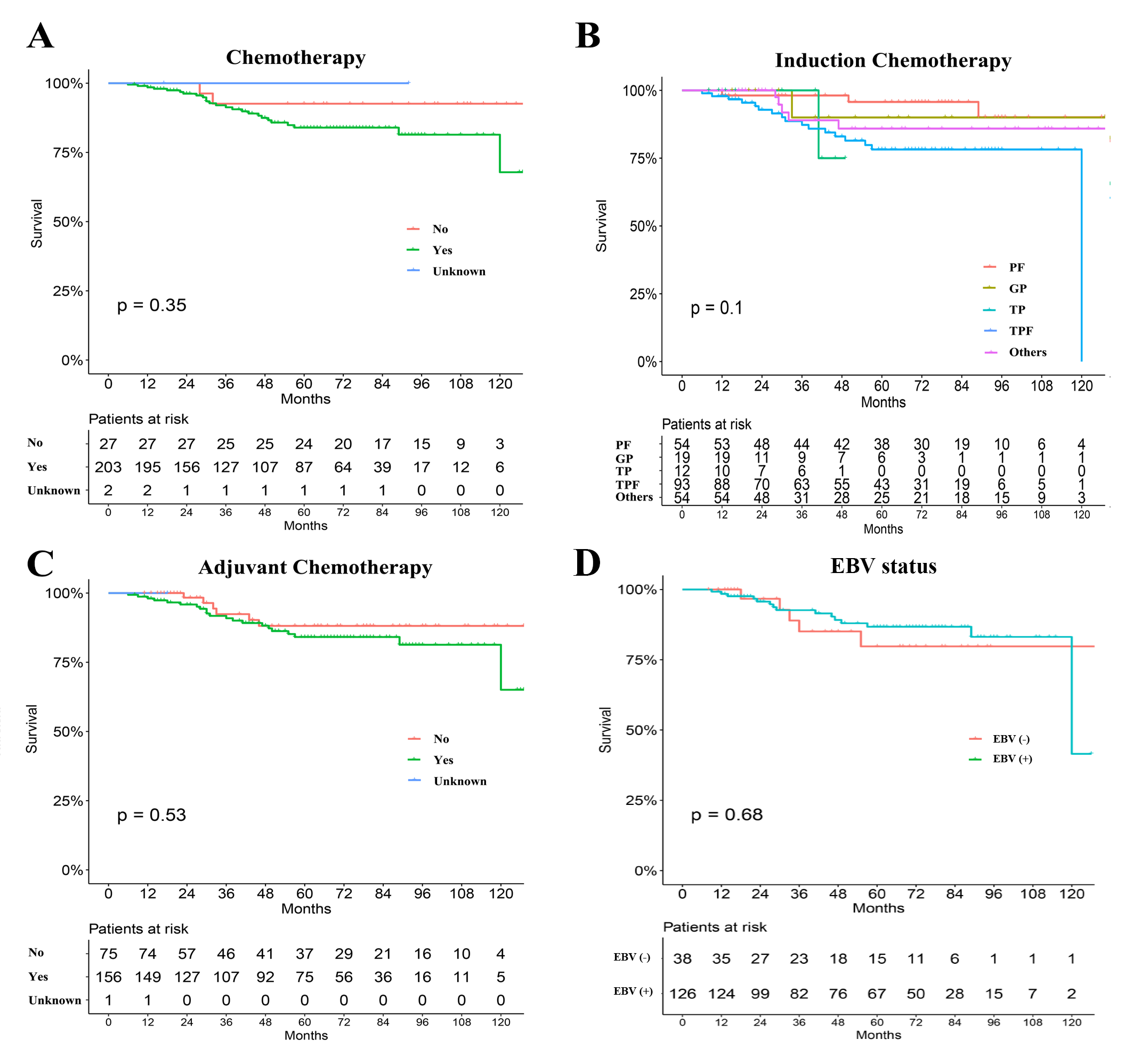

Supplement: Figure S4 — Kaplan-Meier estimates of OS by chemotherapy (A), induction chemotherapy (B), adjuvant chemotherapy (C), and EBV status (D) with NPC from our center. [file Image_4.TIF]
